# Can we take the pulse of environmental governance the way we take the pulse of nature? Applying the Freshwater Health Index in Latin America

**DOI:** 10.1007/s13280-020-01407-8

**Published:** 2020-11-15

**Authors:** Derek Vollmer, Maíra Ometto Bezerra, Natalia Acero Martínez, Octavio Rodríguez Ortiz, Ivo Encomenderos, Maria Clara Marques, Lina Serrano-Durán, Isabelle Fauconnier, Raymond Yu Wang

**Affiliations:** 1 Moore Center for Science, Conservation International, 2011 Crystal Drive, Suite 600, Arlington, VA 22202 USA; 2 Conservation International Colombia, Carrera 13 # 71 – 41, Bogotá, Colombia; 3 Conservation International Peru, Av. Circunvalación N° 1217, Tarapoto, San Martín 22160 Perú; 4 Conservation International Brazil, Av. Rio Branco, 131 - 8th floor - Centro, CEP: 20040-006, Rio de Janeiro, RJ Brazil; 5 Centro del Agua para América Latina y Caribe, Instituto Tecnológico de Monterrey, Carrera 30 # 11-89, 660003 Pereira, Colombia; 6 Global Water Programme, International Union for the Conservation of Nature, Gland, Switzerland; 7 Department of Public Administration, Sun-Yat Sen University, No. 1088 Xueyuan Avenue, Nanshan District, Shenzhen, 518055 P.R. China

**Keywords:** Governance, Indicators, Latin America, Rivers, Stakeholders, Water resource management

## Abstract

Quantitative assessments have long been used to evaluate the condition of the natural environment, providing information for standard setting, adaptive management, and monitoring. Similar approaches have been developed to measure environmental governance, however, the end result (e.g., numeric indicators) belies the subjective and normative judgments that are involved in evaluating governance. We demonstrate a framework that makes this information transparent, through an application of the Freshwater Health Index in three different river basins in Latin America. Water Governance is measured on a 0–100 scale, using data derived from perception-based surveys administered to stakeholders. Results suggest that water governance is a primary area of concern in all three places, with low overall scores (Guandu-26, Alto Mayo-38, Bogotá-43). We conclude that this approach to measuring governance at the river basin scale provides valuable information to support monitoring and decision making, and we offer suggestions on how it can be improved.

**Electronic supplementary material:**

The online version of this article (10.1007/s13280-020-01407-8) contains supplementary material, which is available to authorized users.

## Introduction

Water security rightly ranks as a top environmental concern, and has spurred numerous efforts to accurately measure the quantity, quality, and ecological integrity of freshwater supplies at multiple spatial scales, and for a variety of audiences (Vollmer et al. 2016). But there has also been increasing recognition that issues of water insecurity are generally crises of *governance*, not just problems of inadequate supply or climate variability (Rogers and Hall 2003; McDonnell 2008; Tortajada 2010; Bakker and Morinville 2013; Akhmouch 2014). Water resource management increasingly refers to managing relations among stakeholders, rather than a single institution managing a physical resource (Falkenmark 2004). Where the underlying governance system is weak, stakeholders are unable to efficiently and effectively respond to pressures like pollution, increasing water demand, and freshwater ecosystem degradation. Yet prevailing assessments of sustainability have typically focused on the technical and biophysical factors that readily lend themselves to quantification—these could be viewed as the “outcomes” of water governance (Wiek and Larson 2012; Schneider et al. 2014) but by themselves do not offer insights into the impact of different aspects of governance. Quantitative indicators are now widely used to assess the sustainability or resilience of freshwater systems (Vollmer et al. 2016), but Pires et al’s (2017) review of 170 sets of water sustainability indicators revealed that only about one-third even included governance indicators. And comparative water governance is a growing field, but is dominated by qualitative, one-off assessments (Özerol et al. 2018).

From these existing efforts to quantitatively assess water governance, a number of insights have emerged. The first is that water governance should no longer be viewed as the domain of governments alone; it involves multiple levels of participation from public institutions, along with private sector and civil society actors (Tortajada 2010). The second is that, while “good governance” is a laudable goal, there is no universal definition (Özerol et al. 2018), though guidance has tended to focus on common themes such as the enabling environment (policies, laws, regulations, norms), engagement or participation, and performance (Bertule et al. 2017). The third insight is that, for quantitative analysis there are few alternatives to survey data, including perception-based surveys, but these can provide value in governance assessments (Kaufmann et al. 2010). Consequently, analysts have often resorted to articulating frameworks and general principles for evaluation, rather than normative approaches to quantitative assessment (Woodhouse and Muller 2017), allowing some space for local adaptation and interpretation.

The OECD’s Principles on Water Governance (Akhmouch et al. 2018) is one of the latest examples of this, offering a list of 36 input and process indicators along with a check list for self-assessments. Some frameworks have instead focused on problem structuring and comparative analysis rather than prescribing specific processes, noting that the link between “good” governance processes and improved biophysical outcomes is largely unproven (Knieper et al. 2010; Pahl-Wostl et al. 2010). Others have concentrated more narrowly on specific institutional arrangements, chiefly river basin organizations (RBOs), offering suites of indicators to assess institutional performance (e.g., Hooper 2010) as a proxy for progress toward more integrated water resource management (IWRM). The advent of the Sustainable Development Goals (SDGs) has also renewed interest in measuring water governance at a national and ultimately global scale. Bartram (2018) assesses SDG 6, the “water goal”, and highlights the challenges of linking the governance (“means of implementation”) targets with the more concrete “outcomes” that can be measured in biophysical or economic terms, questioning their universal applicability and acceptability. Bertule et al. (2018) note that the IWRM target (6.5.1) is typically measured via survey administered to a national focal point, often a single person within a Ministry of Water Resources, with three quarters of countries not undertaking stakeholder consultations prior to reporting. Moreover, national level governance indicators, although useful for a quick international comparison, do not necessarily correspond well to environmental and natural resource issues which typically have important local (sub-national) and basin-specific characteristics (Barrett et al. 2006).

**Fig. 1:**
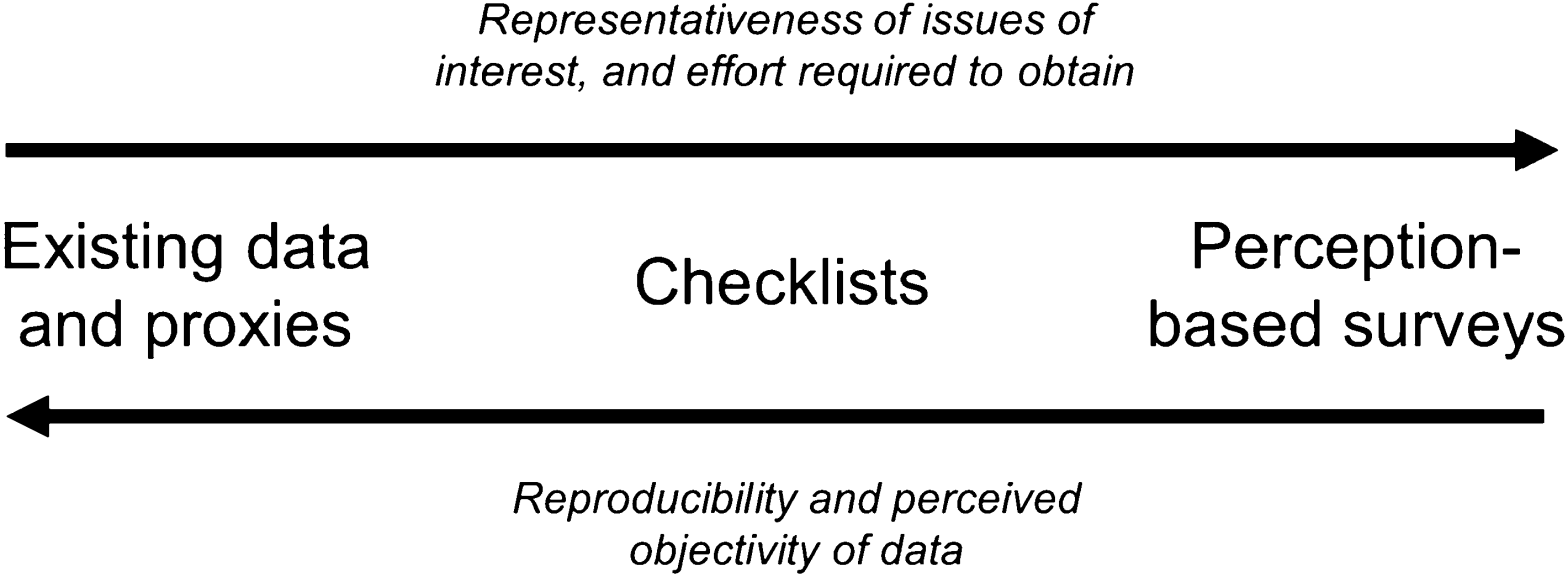
Sources of data for governance assessments, and their relative merit

Analysts of water governance must choose among three possible data sources and collection methods (Fig. 1). Using existing data carries the advantages of transparency, presumed objectivity, and reproducibility (assuming that the datasets are routinely updated). However, suitable data are rarely available, leaving analysts to select between coarse global products such as the Worldwide Governance Indicators (Kaufmann et al. 2010) or the IWRM data portal (UNEP-DHI 2018), or developing numeric proxies such as budget line items, meetings held, or negative media mentions—these are easy to count but are not necessarily relevant to the real subject of interest (Stefano 2010). Moreover, observable data provide a narrow frame for examining the complexity of governance (Olsson and Jerneck 2018). For these reasons, checklists are frequently developed and employed (Wilde et al. 2009); they are designed to facilitate specific data collection in a transparent and repeatable manner. At their simplest, they are constrained to binary choices so that the analyst simply answers yes/no questions and can tally a quantitative score based on that.

However, this is less suitable for monitoring progress (Wilde et al. 2009), and is not designed to capture the nuance or subjectivity of, for example, terms like transparency and accountability (Huitema and Meijerink 2017), or more broadly, the gap between *de jure* and *de facto* governance in a place (Wilde et al. 2009; Kaufmann et al. 2010). Checklists also tend to be rigid, mirroring a noted shortcoming of prescriptive IWRM approaches (Ait Kadi 2019). For example, stakeholder engagement is widely promoted, but still lacks an evidence base demonstrating its impact on water governance (Akhmouch and Clavreul 2016) and may not be universally accepted as a high priority. This has led more assessments toward perception-based data, applying methods ranging from a single analyst using a numeric rating scale (Araral and Yu 2010; Davis et al. 2013) to large groups of stakeholders ranking statements or giving their own ratings (Adger et al. 2005; Carmenta et al. 2017; Cradock-Henry et al. 2017). Though subjective and potentially less reliable than directly observable phenomena (Stefano 2010), perception data are valid because agents base their actions on perception (Kaufmann et al. 2010; Carmenta et al. 2017). Assessing stakeholders’ perceptions can also reveal their current understanding of the governance structure and dynamics (Musacchio et al. 2020).

It is a non-trivial task to “take the pulse” of water governance the way that we now commonly measure nature or the economy, by constructing quantitative indicators that can be monitored over time. Analysts must be cognizant of the tension between local relevance versus global interest, and subjective versus objective information, as well as the logistical challenges of collecting useful data. We build on recent efforts in this field and present an application of the Freshwater Health Index (FHI) framework (Vollmer et al. 2018) to assess water governance in three case studies in Latin America. The FHI includes a composite set of indicators to measure the ecological integrity, delivery of ecosystem services, and governance in river and lake basins. To calculate the governance indicators, we administered a survey to groups of stakeholders in three countries—Guandu basin in the state of Rio de Janeiro, Brazil, Alto Mayo basin in the Andean Amazon region of Perú, and the water supply area for metropolitan Bogotá, Colombia. These same stakeholders underwent a weighting exercise to determine levels of consensus around priorities and, by extension, areas of disagreement that could forestall action on improving water governance. By comparing results from these three studies, we are able to provide insights into how a systematic framework can be applied and adapted locally to measure water governance, the types of information it can generate, and the strengths and weaknesses of the approach.

## Materials and Methods

### Case Study Basins

The three selected case studies all come from South America, where water resources are generally abundant but where water governance, according to a recent country-level assessment, has underperformed relative to other parts of the globe (Bertule et al. 2018). All three basins were part of a project to apply the Freshwater Health Index (Vollmer et al. 2018; Bezerra et al. 2020) and had been selected based on existing relationships with relevant governance stakeholders and not with a goal of cross-basin comparative analysis in mind. At the time of writing we were not aware of any qualitative assessments of water governance in these basins, making it difficult to validate our quantitative results against other assessment methods. However, to the extent possible we interpret survey scores in light of the context of each place.

In the Bogotá, top water management concerns include meeting water demands for the city of about 10 million people, and ensuring that upstream activities such as potato cultivation, tanneries, and artisanal mining do not jeopardize water quality. Water supply is sourced from a system of five watersheds and storage reservoirs, which deliver water to the city and surrounding municipalities, with wastewater then channeled to the Bogotá River. Day to day management is a collaboration among the district government, the water and sanitation public utility known as Acueducto, and the regional environmental agency (CAR). The three entities signed a legal document in 2007 (Convenio 171) that spelled out their respective responsibilities. CAR is to focus on conservation and reversing environmental degradation, executing national policies. Acueducto (which is owned by the District government) focuses on water supply, distribution, and sanitation infrastructure, including wetland protection as a form of natural infrastructure.

The Guandu basin in Brazil is a highly engineered coastal watershed that acts as the water supply to approximately 9 million people in metropolitan Rio de Janeiro, mainly through a diversion of water from the much larger Paraíba do Sul basin (González-Bravo et al. 2019). Most water users reside east of Guandu basin, in neighboring Guanabara basin. The State of Rio de Janeiro created a basin committee (Comite Guandu) in 2002, affiliated with the State Council of Hydrologic Resources, to provide advisory services, lead deliberations, and promote participatory management. Complementing the committee is AGEVAP, its executive arm, which collects user fees and applies the funds to carry out water resource management plans. Top concerns include water quality due to the industrial activities located in Guandu basin, and water allocation between users residing in Guandu and the larger urban population in the city of Rio de Janeiro.

Finally, the Alto Mayo basin in Perú is a typical Andean-Amazonian watershed, still nearly 80% forested but experiencing some of the highest rates of deforestation in the region as land is cleared for livestock, rice, and coffee cultivation. The basin’s population of 248,000 people includes 14 indigenous communities with customary rights over one-fifth of the area. Compared to the other two case study basins, water governance in the Alto Mayo basin is more centralized—the national government has traditionally played a strong role focused on irrigation development, although a basin committee was recently and voluntarily established as the first of its kind in the Andean Amazon (ANA 2017). A substantial portion of the basin is protected forest, and although water supply is abundant, concerns are emerging about water quality due to excess sediment from forest degradation, and pollution from coffee processing.

In all three basins, a much wider network of stakeholders play important roles in water governance, including environmental monitoring, territorial planning, biodiversity conservation, advocacy, and women’s empowerment. We do not detail all of those entities here but all were involved in the assessment process. This is one of the aims of the methods we present, to represent voices from these diverse water governance actors.

### FHI Governance and Stakeholders assessment

**Fig. 2:**
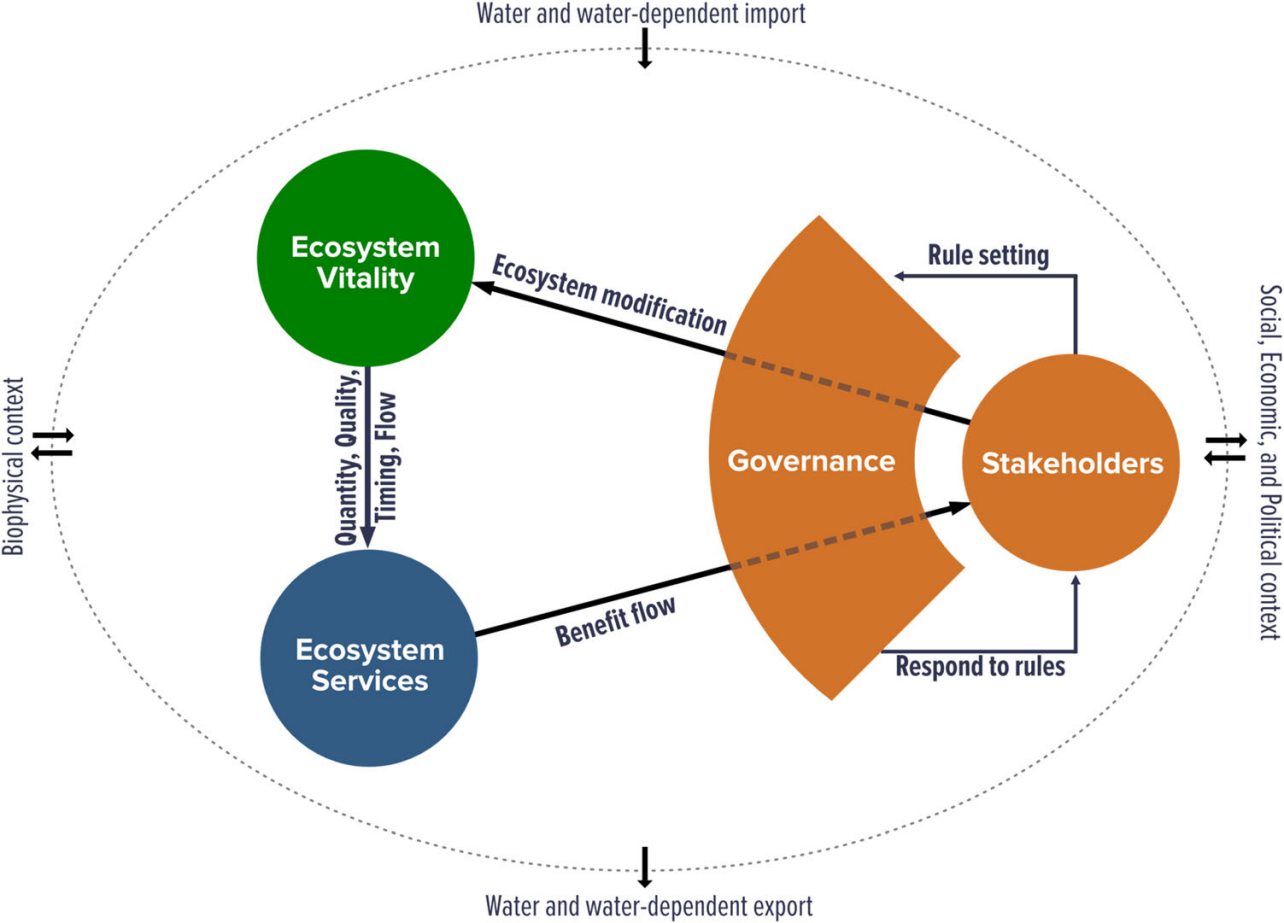
Freshwater Health Index conceptual framework (from Vollmer et al. (2018) from *Science of the Total Environment*)

Between March and November 2018, a team of researchers worked with stakeholders from each of these basins to apply the FHI framework. The full framework (Fig. 2) includes indicators for ecological integrity, ecosystem services, and governance, and has been applied in China (Vollmer et al. 2018) and the Lower Mekong (Liu et al. 2019; Souter et al. 2020). This is the first application of the framework in Latin America, and the full case studies and biophysical results are presented in Bezerra et al. (2020). FHI indicators are all quantified and placed on a 0–100 scale for ease of comparison—governance indicators are not combined with biophysical indicators to create a greater composite, but the rationale behind providing quantitative indicators is so that all aspects of the freshwater system can be monitored over time, according to the common framework, and priorities identified. It is not designed for inter-basin comparison mainly because the target end-users are decision makers within each basin.

Here for the first time we present the detailed methods applied in measuring the “Governance & Stakeholders” pillar of the Index. This pillar comprises four major indicators that further comprise twelve sub-indicators (see Table 1). The Governance & Stakeholders indicators share common elements with the OECD’s principles of water governance (Akhmouch et al. 2018) as well as the earlier UNDP guidance on assessing water governance (Jacobson et al. 2013), such as:

**Table 1:** Major and sub-indicators of water governance used the in the Freshwater Health Index assessments (Vollmer et al. 2018)

Major indicator	Sub-indicator
*Enabling environment*: the policies, regulations, market mechanisms and social norms used in managing freshwater resources, and the human and financial resources available	*Water resource management*: degree to which institutions are performing key management functions such as coordination, planning and financing, and conflict resolution
	*Rights to resource use*: existence and clarity of rules as well as rights pertaining to water use, allocation, pollution, and related basin resources
	*Incentives and regulations*: availability of different management instruments, such as impact assessments and economic incentives
	*Technical capacity*: number and skill level of professionals working in water resource management
	*Financial capacity*: investment gap between allocated and required finances for water resource protection measures
*Stakeholder engagement*: the degree of transparency and accountability around stakeholder involvement in basin decision-making	*Information access and knowledge*: accessibility of data on water quantity, quality, resource management and development
	*Engagement in decision*-*making processes*: scope of stakeholder involvement and degree to which they have a voice in the cycle of policy and planning
*Vision and adaptive governance*: the capacity to apply new information to develop and adjust both policies and plans for the basin	*Strategic planning and adaptive governance*: degree to which comprehensive strategic planning at the basin or sub-basin scale take place
	*Monitoring and learning mechanisms*: quality and use of physical, chemical, biological, and socioeconomic data to guide policy and planning
*Effectiveness*: the outcomes of water-related policies and investment decisions	*Enforcement and compliance*: degree to which laws and related agreements are upheld and enforced
	*Distribution of benefits from ecosystem services*: degree to which different segments of the population are positively impacted by decisions about water resource management
	*Water*-*related conflict*: presence of conflicts over allocation, access, pollution, diversion or infrastructure development

- focusing on the broader governance context in which specific paradigms such as IWRM operate,
- including stakeholders as a variable within governance, and
- accounting for effectiveness (in addition to process).

In addition, the FHI framework includes a major indicator and two sub-indicators that focus on the idea of adaptive governance (Bakker and Morinville 2013; Chaffin et al. 2014).

We administered a perception-based survey to groups of representative stakeholders in each basin, with the goal of creating a richer dataset that reflects the diversity of opinions in each governance system. We used purposive sampling to identify participants, by mapping the stakeholder groups in each basin and inviting a cross-section to ensure participation from the various levels and agencies of government, along with participants from civil society, academia, and the private sector.

Using a Likert-type ordinal (1–5) rating scale, participants were asked to rate their opinion regarding the quality or degree of implementation of a variety of topics related to water governance in their respective basins. The survey instrument was written first in English (Supplementary Material) and then translated into Spanish and Portuguese. It included 12 modules, each corresponding to a sub-indicator (Table 1), and each module contained between three and six statements for participants, totaling 51 statements, though everyone was instructed to only rate statements for which they felt knowledgeable. Surveys were administered in person at separate workshops (one per basin). Responses were kept anonymous, although respondents’ sectoral affiliation (e.g., government, or civil society organization) and geographic location (e.g., upstream or downstream) were recorded. In total, 22 respondents completed the survey for Guandu basin, 29 for Alto Mayo, and 60 for Bogotá—the latter had a larger sample to reflect its larger geographic area and the fact that the study area actually comprises five different sub-basins and thus a greater diversity of local stakeholder groups.

Data were aggregated in each basin for the purposes of calculating mean values for each of the 12 sub-indicators, the four major indicators, and a final summary value for the Governance & Stakeholders component of the FHI assessment. Although it is generally inadvisable to take the mean of ordinal scale data because the difference between points are not necessary equal, individual items can be grouped into constructs, or “survey scales”, such as our 12 modules, and then taking the mean of these constructs is appropriate (Sullivan and Artino 2013). We calculated Cronbach’s alpha (Eq. 1) for each sub-indicator, to evaluate whether the individual statements or components of each scale were intercorrelated and thus a reliable or internally consistent measure.

1 $$ \alpha = \left( {\frac{k}{k - 1}} \right)\left( {1 - \frac{{\mathop \sum \nolimits_{i = 1}^{k} \sigma_{{y_{i} }}^{2} }}{{\sigma_{x}^{2} }}} \right) $$

where: *k* is the number of scale items, $\sigma_{{y_{i} }}^{2}$ is the variance associated with item *i,* $\sigma_{x}^{2}$ is the variance associated with the observed total scores

If the scale items have no covariance, *α* would = 0; as covariance increases, *α* approaches 1. Although there is no strict guideline for interpreting *α*, the typical minimum recommended coefficient value is between 0.65 and 0.8 for scales with a small number of components or test items (Vaske et al. 2017) such as our survey modules. Finally, we calculated the interquartile range (IQR) for each of the 51 statements. This provides a measure of the variance of responses, as an accompaniment to the mean value, in cases where responses might be polarized around extreme values rather than exhibiting a normal distribution. We therefore use the IQR as a measure of consensus (Novakowski and Wellar 2008), with values ≤ 1.0, 1.01–1.99, and ≥ 2.0 corresponding to “high”, “moderate”, and “low” consensus, respectively. But the IQR also allowed us to test what effect, if any, increasing our sample size had in reducing variance, which could in turn increase confidence in the results and lessen the impact of any one respondent registering extreme values.

### Weighting and scoring indicators

The 51 individual statements were left unweighted, meaning that, for example, if a module had three questions, each statement contributed one-third of the average rating for the corresponding sub-indicator. But because sub-indicators were aggregated into the four major indicator scores, and those were subsequently aggregated to a final Governance & Stakeholders value, we asked participants to undertake a weighting exercise so that the four indicators and their twelve sub-indicators could be weighted in a transparent manner specific to that particular basin. To avoid our survey results biasing participants’ views of what they consider more or less important, we administered the weighting exercise prior to sharing any results from the governance survey. For this, we used the Analytic Hierarchy Process (AHP) (Saaty 2005), in which participants made a series of pairwise comparisons first among the major indicators (the top-level hierarchy), and then within each major indicator group, among its sub-indicators. Participants used a standard linear (1-9) scale to register their individual preferences. These quantitative preferences fill a comparison matrix, from which we calculate the normalized Eigen vector, giving us each individual’s relative weights or priorities, along with a consistency ratio. Global (weighted geometric mean) priority weights are calculated for the group, along with a consistency ratio for the group and a consensus measure, based on Shannon *α* and *β* entropy (Goepel 2013). Again, there is not a strict standard for measuring group consensus, but in this case we interpret 65% and below as *low* consensus, 65–75% *moderate*, and greater than 75% to be *high*.

To calculate final scores on a 0–100 scale, we first averaged the ratings for each of the 12 modules. Since these ratings ranged between 1 and 5, we transformed the scale by subtracting 1 from the average rating and multiplying the result by 25 to arrive at a score out of a possible 100. As a reference, a rating of 3 corresponds to a numeric score of 50. To aggregate these sub-indicator scores into major indicator scores, we used the stakeholder-derived weights and calculated a weighted geometric mean, i.e., $x_{1}^{{w_{1} }} \times x_{2}^{{w_{2} }} \times \cdots x_{n}^{{w_{n} }}$, which is more sensitive to the weights than an arithmetic mean. We performed the same process on the major indicators to arrive at an overall score.

## Results and discussion

The Freshwater Health Index was designed for use in monitoring and resource management in individual basins, not necessarily to promote comparative analyses across case studies, given the unique geographic, hydrologic, and sociopolitical contexts of basins around the world (Vollmer et al. 2018). Thus, we present here the results of the governance assessments from the three cases to illustrate the insights that our methods can produce and their applicability in a range of contexts. Among the three basins, Guandu registered the lowest overall score with 26 (out of 100), followed by Alto Mayo at 38 and Bogotá at 43. It is apparent that overall, water governance is not performing up to most stakeholders’ expectations in any of the basins and so a general conclusion we can draw is that improving water governance should be a high priority in all three places. But the exceedingly low score in Guandu was surprising, given that it appears to have the most mature formal governance structure. This may be owing to the fact that most of its water resources are derived from an interstate basin (Paraiba do Sul, shared between Rio and Sao Paulo states), and most of its beneficiaries reside in another basin (Guanabara), but stakeholders also noted that there is a gap between the existence of formal mechanisms and their successful implementation. This is also consistent with the observation that the implementation of IWRM principles has been very slow throughout Brazil, in spite of the creation of river basin committees (Costa et al. 2017). Table 2 summarizes the full results of both the governance survey and the weighting exercise. We found that the Cronbach’s alpha was satisfactory for each sub-indicator, with values ranging from 0.62 to 0.95, and averages of 0.80 for Alto Mayo and Bogotá and 0.82 for Guandu, suggesting that our groups of statements and survey instrument overall are suitable measures.

**Table 2:** Indicator scores and priority weights for all three basins

Major indicator	Weight	Score	Sub-indicators	Weight	Score
*Guandu*					
Enabling Environment	0.11(L)	26(M)	Water resource management	0.14(M)	42(L)
			Rights to resource use	0.23(M)	26(M)
			Incentives & regulations	0.22(M)	29(M)
			Technical capacity	0.21(M)	27(M)
			Financial capacity	0.19(M)	20(H)
Stakeholder Engagement	0.25(L)	25(M)	Information access and knowledge	0.60(L)	27(M)
			Engagement in decision-making processes	0.40(L)	22(M)
Vision & Adaptive Governance	0.41(L)	24(M)	Monitoring mechanisms	0.65(L)	19(H)
			Strategic planning and adaptive governance	0.35(L)	35(M)
Effectiveness	0.24(L)	28(M)	Enforcement and compliance	0.27(L)	23(H)
			Distribution of benefits from ecosystem services	0.35(L)	23(H)
			Water-related conflict	0.38(L)	37(M)
*Alto Mayo*					
Enabling Environment	0.12(L)	38(M)	Water resource management	0.27(L)	40(M)
			Rights to resource use	0.12(L)	36(H)
			Incentives & regulations	0.11(L)	49(M)
			Technical capacity	0.26(L)	39(H)
			Financial capacity	0.24(L)	30(M)
Stakeholder Engagement	0.40(L)	40(M)	Information access and knowledge	0.47(L)	44(M)
			Engagement in decision-making processes	0.53(L)	37(H)
Vision & Adaptive Governance	0.28(L)	37(H)	Monitoring mechanisms	0.36(L)	34(M)
			Strategic planning and adaptive governance	0.64(L)	38(H)
Effectiveness	0.20(L)	35(M)	Enforcement and compliance	0.40(L)	35(M)
			Distribution of benefits from ecosystem services	0.41(L)	31(M)
			Water-related conflict	0.19(L)	46(M)
*Bogotá*					
Enabling Environment	0.23(L)	42(M)	Water resource management	0.22(L)	43(M)
			Rights to resource use	0.12(L)	48(M)
			Incentives & regulations	0.15(L)	57(M)
			Technical capacity	0.23(L)	39(H)
			Financial capacity	0.26(L)	40(M)
Stakeholder Engagement	0.29(L)	44(M)	Information access and knowledge	0.47(L)	45(M)
			Engagement in decision-making	0.53(L)	43(M)
Vision & Adaptive Governance	0.25(L)	42(M)	Monitoring mechanisms	0.55(L)	42(M)
			Strategic planning and adaptive governance	0.45(L)	41(H)
Effectiveness	0.26(L)	43(H)	Enforcement and compliance	0.32(L)	42(M)
			Distribution of benefits from ecosystem services	0.34(L)	46(H)
			Water-related conflict	0.34(L)	41(H)

Measures of consensus on each indicator and weight are indicated with a L (low), M (moderate), or H (high)

With such small sample sizes (ranging from *n *= 22 in Guandu to *n *= 60 in Bogotá), we caution against interpreting too much from split samples (in this case, by sector). But qualitatively, we did observe trends in Alto Mayo and Bogotá that conformed to hypotheses, namely that government representatives were more likely to provide scores greater than the mean, while NGO and community representatives provided scores lower than the mean, with the “experts” from academia splitting the difference. This trend was most pronounced in Bogotá, where we compared the responses of those from the public sector (*n *=34) with all other groups (academia, industry, NGOs, and community groups, *n *= 26). Recalculating scores with the government subset yielded an overall score of 51, with sub-indicator scores of 54, 53, 45, and 51. The other sample of all non-government stakeholders yielded an overall score of 36, with sub-indicator scores of 40, 35, 37, and 32, highlighting a substantial difference between government stakeholders’ perception and that of the non-government stakeholders. When we singled out community and NGO actors, their averages were even lower than the non-government group mean (32 and 34 respectively). Finally, we ran one-tailed t-tests, assuming unequal variance, on responses for each of the 51 statements and found that the two groups (government and non-government) were statistically different (*p* < 0.05) on 35 of them. We must reiterate that these are *perception* data, thus there is not an objective true rating, but in cases like this, the differing mean values between sub-groups highlight discrepancies between stakeholder groups, and at a minimum these discrepancies are fodder for further discussion and analysis.

### Guandu, Brazil

For the *Enabling Environment* category, stakeholders in Guandu placed a comparatively low weight (relative to stakeholders in Alto Mayo and Bogotá) and registered the highest score for the *Water Resource Management* sub-indicator, and placed comparatively higher weights on both *Rules for Resource Use* and *Incentives and Regulations*. This perhaps reflects the fact that the laws about roles and responsibilities for river basin committees are already promulgated; participants also gave the highest overall rating (mean of 3.1 on the 1–5 scale) on the statement relating to infrastructure being centrally managed. The quality and clarity of rules around water allocation received a relatively high rating (2.7), while rules for groundwater abstraction were rated the lowest in that sub-indicator category (1.8), similar to the situation in neighboring Sao Paulo (Borges and Santos 2014). Ratings for financial incentives for environmental stewardship, as well as land use zoning policy, were high (2.7), but market-based schemes rated low (1.6), reflecting the fact that there has been little consideration of, for example, tradeable water rights in the region. Stakeholders placed a high weight on the sub-indicator *Water*-*related conflict*, which received the overall highest sub-indicator score in Guandu at 37 and seems to correlate with the higher rating for rules around allocation. The score was driven by a relatively high rating (meaning good performance) for conflicts about water rights allocation (3.0) but a low rating for conflicts regarding water access (1.8). It was explained that, in particular, residents in Rio municipality typically enjoy reliable access to water services, while residents of the municipalities actually living in Guandu basin experience inferior service. Stakeholders remarked that the FHI as implemented did not seem to capture this discrepancy adequately, particularly for the four municipalities physically located within Guandu basin (Queimados, Paracambi, Japeri, and Nova Iguaçu) that have been subject to curtailed water supplies 2–3 times per week, as a result of switchover operations (Britto et al. 2016). Finally, stakeholders placed their highest weight on the *Vision and Adaptive Governance* indicator, and a high weight on the *Monitoring and Learning Mechanisms* sub-indicator, which actually received the lowest score (19) of the entire governance assessment. As a result, stakeholders became more aware of the quality and coverage of existing data, the lack of reliable information on climate and discharge in particular, and the fact that not all existing monitoring stations are actively collecting data. Thus, one initial outcome of the FHI assessment was that stakeholders from the Guandu Committee and AGEVAP stated that they would use the results to guide investments in additional monitoring.

### Alto Mayo, Peru

Despite being a remote watershed in the Andean Amazon with a comparatively small population, the Alto Mayo has been a pilot site for numerous water governance initiatives. But this small population and thus lack of a revenue base of water users may help explain why the five statements about financial capacity garnered the lowest average score (2.2 out of a possible 5, where 3 corresponds to “Satisfactory”). Among these, water supply and delivery system investments scored marginally higher (2.5 and 2.6), while wastewater management, ecosystem conservation, and monitoring and enforcement investments all scored 2.0 or lower. This gap between policy and investment is also illustrated by the fact that *Incentives and Regulations* received the highest sub-indicator score (49), while *Financial Capacity* received the lowest (30). Given the amount of forest conservation and agriculture taking place, and the income inequality between upstream residents and the more urbanized downstream communities, Alto Mayo has been testing out payments for watershed services as an additional financial mechanism since residents in the city of Moyobamba agreed to an increase in fees in 2009 (Stern and Echavarria 2013). Perhaps the most interesting result from Alto Mayo was the high weight stakeholders assigned to the *Stakeholder Engagement* indicator (0.4). The region is home to a large population of indigenous peoples and the corresponding score for this indicator (40) outperformed the overall score slightly, but the score for the *Information Access* sub-indicator was much higher than its companion sub-indicator on *Engagement in Decision*-*making Processes* (44 versus 37). Stakeholders placed their lowest weight on the *Water*-*related Conflict* sub-indicator (0.19), which incidentally received the highest sub-indicator score (46), suggesting that it is at present a lower concern. Interestingly, however, the statements in the *Water*-*related Conflict* sub-indicator exhibited the highest average IQR (1.85) of any sub-indicator we evaluated across all three basins. Statements about overlapping jurisdictions, water access, infrastructure siting, and downstream water quality conflicts all had an IQR of 2, signifying “low” consensus. One factor underpinning this could be that there is also a perception that the most economically vulnerable populations in the Alto Mayo basin are not equally benefitting from the region’s resources, as stakeholders rated that statement lowest of all (1.9) and with a relatively low IQR (1) and variance (0.7) in responses. This is consistent with findings in Ostovar (2019), where Peru’s historically marginalized communities in the Andes hold distinctly different preferences and worldviews regarding watershed protection, relative to the majority of downstream users.

### Bogotá, Colombia

Compared to the other two basins, stakeholders in Bogotá placed greater emphasis on the *Water Resource Management*, giving it a weight about double (0.23) that observed in Guandu and Alto Mayo. Among the individual statements there, the (translated) statement “Ecosystem conservation priorities are developed and actions implemented” was rated especially low (2.3). Its best performing sub-indicator was *Incentives and Regulations*, with a score of 57, but this was brought down by a low rating for honorary recognition programs (2.5) although this statement also had the highest IQR (2.5), suggesting either strong disagreement about the quality of such programs (the majority of respondents rated a 1, meaning they either do not exist or are in an early stage of discussion) or confusion about what types of programs would belong in this category. Another statement that stood out in the Bogotá assessment was the rating on gender, specifically how women and girls benefit from ecosystem services. Stakeholders rated the statement 3.2, the highest of all individual statements in the survey, and nearly a full point higher than Guandu and Alto Mayo (both 2.3). Finally, water quantity monitoring was rated relatively high (3.1), which is not surprising given the role that the municipal utility plays in managing water supply for the region. On the other hand, biological and ecological monitoring received the single lowest rating (2.3) of any statement on the Bogotá assessment, and was thus noted as a priority area for improvement.

### The utility of governance “self-assessments”

One major distinction of the approach presented here is that governance actors themselves lead the assessment (as opposed to an external analyst), and the methods can flexibly accommodate as many actors as are interested in participating. Granted, we as analysts introduced the framework and administered surveys, not unlike qualitative researchers conducting interviews and document reviews. But by relying solely on perception data and standardized responses, remaining biases in the analysis are largely attributable to the respondents themselves, i.e., those who represent the water governance system in each basin, rather than the external analyst. Had we only surveyed academic experts, we might have obtained similar quantitative scores, at least in the cases of Alto Mayo and Bogotá, but we would not have been able to observe the slight positive and negative biases that government and non-government actors, respectively, hold. This is important in terms of the transparency of our approach but is also potentially important information for the governance actors themselves as they work toward more participatory and integrated water resource management. After all, they base decisions on their perception (Kaufmann et al. 2010; Carmenta et al. 2017) and so it is helpful to have more insight into what perceptions are and how they vary among stakeholders.

Similarly, the weighting exercise gives agency to the actors themselves to identify their preferences and, by extension, their priorities when it comes to maintaining or improving freshwater health. Indicator-based assessments do sometimes allow the decision makers to weight components, but there is not a universal process for doing so, particularly in group settings (Sharpe 2004; Vollmer et al. 2016). We employed the commonly used AHP but a range of weighting approaches from decision science (e.g., rating, ranking, ratios) would suffice. Based on our results, as measured by consistency ratios, and subsequent feedback from participants, the pairwise rating scheme of the AHP was challenging, particularly with four or more sub-indicators, because the required choice sets and thus chances for inconsistent responses are much greater. AHP software typically offers prompts to participants to adjust their responses if they fail to meet a prescribed consistency ratio, but this is not a fail-safe. For this reason, we suggest exploring other methods that are slightly less cognitively demanding but still allow for maximum participation.

The survey instruments we developed provide a template for decision makers in each basin to monitor changes over time. This may help to raise the profile of water governance on par with the more regular monitoring of the biophysical indicators of freshwater health. And as we found in all three basins, the governance indicators are almost universally low-performing, meaning that there is a lot of room for improvement and therefore a need to monitor this improvement. Having a baseline measure to compare against can also help decision makers understand how far the governance system needs to improve and, by extension, how long this might take. Changes in the governance system take time and are typically non-linear (e.g., a new water law’s passage could have substantial and far-reaching impacts), so it is too soon to say the right frequency for this kind of monitoring, or what specific factors can drive changes in indicator scores. Still, with the baseline established and tools in hand, these data will eventually be available and valuable in future research. Although the results of these surveys are influenced by the stakeholders who participate, by involving a broad group of stakeholders they should be less susceptible to bias than assessments done by a single analyst or small group. The results from Bogotá showed how non-government actors provided a “counter-weight” to government actors; an assessment excluding one of these sub-groups would have presented a different picture.

### Issues of scale, inclusion and consensus

Water governance in the three case studies, as is true in most of the world, is becoming more polycentric (Woodhouse and Muller 2017). Rather than specify an ideal scale or key actor with the FHI, it is important for information to flow between spatial scales (Rouillard and Spray 2017), recognizing the influence of power dynamics as well as the multi-level governance processes in place (Bakker and Morinville 2013; Norman et al. 2013). In the case of Alto Mayo, we were working with a watershed committee, but this is still an informal mechanism nested within the larger Huallaga River basin, which is the hydrographic region designated by Peru’s national water agency, ANA. ANA recently conducted its own nationwide water governance assessment, using a checklist and qualitative indicators following the OECD framework, (ANA 2018). The richer and finer scale data we collected here could conceivably be nested within ANA’s broader assessments, and our methods could be replicated nationwide, but would require similar workshops that collectively might involve thousands of participants. The finer scale assessment approach that we have demonstrated supports a primary goal of the FHI, which is to help more stakeholders understand their impacts and dependence on ecosystem services in their particular basin.

Yet the Guandu example demonstrated that single hydrographic regions and their management committees are not necessarily the most suitable scale of assessment either. One of the main recommendations from stakeholders there (many of whom meet as part of the Guandu Basin Committee) is that the assessment needs to be extended to stakeholders from neighboring Guanabara Bay and the middle Paraiba do Sul River Basin, to accurately reflect the water supply and demand areas of metropolitan Rio. Similarly, the Bogotá case study area involves portions of five different hydrographic basins making up its municipal supply system. This is one of the reasons for the larger group of stakeholders in the Bogotá case, as we sought to have representation from all five basins in the supply system, along with representatives from the various communities and local governments.

The Bogotá case also allowed us to test a hypothesis about sample size. Our hypothesis was that by increasing the sample size, we would reduce the variance and IQR of responses, providing additional confidence that scores were not unduly influenced by a small faction. While it is true that the average IQR for Bogotá was the lowest of the three cases (1.17 compared to 1.26 for Guandu and 1.34 for Alto Mayo), there was no clear reduction in variance measured at the sub-indicator level. Moreover, the measures of consensus for indicator weights in the Bogotá case were no different from the other two basins (Table 2). Therefore, we would suggest that the number of participants in the surveys should be determined based on a judgment about stakeholder groups that should have a voice in the assessment process, recognizing that there will be an upper limit to the number of people in a given geography with sufficient knowledge about the range of water governance issues. Representativeness is likely more important than sample size in gaining insight into actual water governance dynamics.

Which leads to another issue—how best to reflect the differing viewpoints that arise during the assessment. The indicators in the FHI, like many quantitative approaches, require a mean or summary value which, in the case of the governance sub-indicators as well as the weights, may not capture the variance in responses. We measured and reported this variance, and of course the lower the variance, the more confidence we have that our mean values are adequately representing the collective perception of the participants. But less than a third of all the weighting tasks the groups completed registered as at least “moderate” consensus (scores of 65 and above). In other words, for the vast majority of reported weights we observed low or very low consensus—participants may be disagreeing on the relative weights or even the ranking of the indicators and sub-indicators. Like the OECD approach, we report on the strength of consensus for each group of indicators, though in the case of the OECD it is qualitative and so it is unclear how it is determined. This contrasts with the current process for measuring SDG 6.5.1, where workshops are encouraged to “foster consensus” around scores (Bertule et al. 2018) but without guidance on what constitutes consensus. Our reporting of IQRs for each sub-indicator suggests that, with a few exceptions, consensus was generally “moderate” or “high” (Table 2).

In this research we treated consensus as a measurable, making clear that the summary scores represented mean values that in some cases were masking highly diverse viewpoints. The goal of the initial FHI assessment is to develop a baseline understanding of the biophysical and water governance dynamics in a basin, not necessarily to force consensus among decision makers as to what these dynamics look like. This informal network of stakeholders is thus actively contributing to the co-production of knowledge of governance in their basin, which should help with the legitimacy of the information (Armitage et al. 2015). By first eliciting their individual responses, stakeholders can understand how divergent their views are and determine whether and where to compromise when it comes to governance in their basin. Individual responses and voices might become muted if there are more dominant actors in the room, or less engaged if the process is treated as a validation of pre-determined (e.g., government or expert-led) scoring. Without this information about the level of consensus and, more importantly, where there may be geographic or sectoral factions, decision makers may be missing opportunities to resolve underlying conflicts or heading off impending ones. In a separate exercise, one might consider adopting a Delphi-method approach, where initial results of our assessment are revealed to participants who are then given the opportunity to debate the issue and amend their responses if they would like to. Stakeholders often benefit from having dedicated time to debate issues not easily captured by data and measurement (Bosch et al. 2012).

## Conclusion

We have designed the FHI approach to water governance assessment to be low-cost and provide this basic, baseline information, as a sort of screening before deeper (more costly and complex) diagnostics. We demonstrated through three case studies how the FHI can be applied in varying contexts. The diversity of perspectives it can accommodate is a strength, but augmenting the quantitative results with more qualitative information is advisable (Wesselink et al. 2017) to provide deeper insights into issues of influence, networks, and power dynamics (McDonnell 2008) and lend further interpretation to the numbers and their nuances. By incorporating stakeholders’ perceptions, there is not going to be a single objective “reality” of the governance system that we might compare our results against. Our measures of consensus illuminate where stakeholders are aligned and where there may in fact be divergent factions. Our results generally conformed to stakeholders’ expectations and previous qualitative studies from the basins, but our assessment provides new and valuable insights. Like an ECG, the FHI does not prescribe treatment. However, if used to monitor changes over time, it can be a valuable tool in adaptive water governance (Pahl-Wostl 2019). And although our focus is on the basins where the tool is being applied, if applied in a standard and transparent way, our framework and results can contribute to knowledge accumulation across cases (Wilde et al. 2009; Özerol et al. 2018). Water governance is increasing in its complexity as well as its importance, and so there is a clear need to harness all the knowledge we can from cases around the world in a way that is systematic yet retains the context of individual systems.

## Electronic supplementary material

Below is the link to the electronic supplementary material.

Supplementary material 1 (PDF 325 kb)

Supplementary material 2 (XLSX 35 kb)

## References

[CR1] Adger WN, Brown K, Tompkins EL (2005). The political economy of cross-scale networks in resource co-management. Ecology & Society.

[CR2] Ait Kadi M, Martinez-Santos P, Aldaya MM, Llamas MR (2019). Integrated water resources management (IWRM): The international experience. Integrated water resources management in the 21st century: Revisiting the paradigm.

[CR3] Akhmouch A (2014). Water governance in OECD countries: A multi-level approach the “water crisis” is largely a governance crisis. OECD Water.

[CR4] Akhmouch A, Clavreul D (2016). Stakeholder engagement for inclusive water governance: “Practicing What We Preach” with the OECD water governance initiative. Water.

[CR5] Akhmouch A, Clavreul D, Glas P (2018). Introducing the OECD principles on water governance introducing the OECD principles on water governance. Water International.

[CR6] ANA (Autoridad Nacional de Agua). 2017. Resolución Jefatural No 153. Informe No 064-2017-ANA-DCPRH/GRH de 23 de mayo de 2017.

[CR7] ANA. 2018. OECD Water governance indicator framework: Peru–Pilot Test. Retrieved August 8, 2019 from https://www.oecd.org/cfe/regional-policy/oecd-water-governance-indicator-framework.htm.

[CR8] Araral, E., and D. Yu. 2010. *Asia Water Governance Index*. Singapore: Institute of Water Policy, Lee Kuan Yew School of Public Policy, National University of Singapore.

[CR9] Armitage D, de Loë RC, Morris M, Edwards TWD, Gerlak AK, Hall RI (2015). Science–policy processes for transboundary water governance. Ambio.

[CR10] Bakker K, Morinville C (2013). The governance dimensions of water security: A review. Philosophical Transactions of the Royal Society A: Mathematical, Physical and Engineering Sciences.

[CR11] Barbosa Costa M, Mushtaq S, Alam K (2017). Integrated water resources management: Are river basin committees in Brazil enabling effective stakeholder interaction?. Environmental Science & Policy.

[CR12] Barrett CB, Gibson CC, Hoffman B, McCubbins MD (2006). The complex links between governance and biodiversity. Conservation Biology.

[CR13] Bartram J (2018). Policy review of the means of implementation targets and indicators for the sustainable development goal for water and sanitation. NPJ Clean Water.

[CR14] Bertule, M., P. K. Bjørnsen, S. D. Costanzo, J. Escurra, S. Freeman, L. Gallagher, R. H. Kelsey, and D. Vollmer. 2017. *Using indicators for improved water resources management Guide for basin managers and practitioners*. 82 pp. ISBN 978-87-90634-05-6. http://ian.umces.edu/pdfs/ian_report_560.pdf.

[CR15] Bertule M, Glennie P, Bjørnsen PK, Lloyd GJ, Kjellen M, Dalton J, Dalton J, Dalton J (2018). Monitoring water resources governance progress globally: Experiences from monitoring SDG indicator 6.5.1 on integrated water resources management implementation. Water.

[CR16] Bezerra, M. O., D. Vollmer, N. Acero, M. C. Marques, D. Restrepo, E. Mendoza, B. Coutinho, I. Encomenderos, et al. 2020. Operationalizing integrated water resource management in Latin America: Insights from applications of the Freshwater Health Index. Preprint. https://www.researchgate.net/publication/344112622_Operationalizing_Integrated_Water_Resource_Management_in_Latin_America_Insights_from_application_of_the_Freshwater_Health_Index. 10.1007/s00267-021-01446-1 PMC9012716 33693960

[CR17] Borges B, Santos M (2014). Water security in the metropolitan region of Rio de Janeiro: Contributions to the debate. Ambiente & Sociedade.

[CR18] Bosch D, Pease J, Wolfe ML, Zobel C, Cobb TD, Osorio J, Evanylo G (2012). Community DECISIONS: Stakeholder focused watershed planning. Journal of Environmental Management.

[CR19] Britto AL, Formiga-Johnsson RM, Carneiro PRF (2016). Water supply and hydrosocial scarcity in the Rio de Janeiro metropolitan area. Ambiente & Sociedade.

[CR20] Carmenta R, Zabala A, Daeli W, Phelps J (2017). Perceptions across scales of governance and the Indonesian peatland fires. Global Environmental Change.

[CR21] Chaffin BC, Gosnell H, Cosens BA (2014). A decade of adaptive governance scholarship: Synthesis and future directions. Ecology and Society.

[CR22] Cradock-Henry NA, Greenhalgh S, Brown P, Sinner J (2017). Factors influencing successful collaboration for freshwater management in Aotearoa, New Zealand. Ecology & Society.

[CR23] Davis C, Williams L, Lupberger S, Daviet F (2013). Assessing forest governance: The governance of forests initiative indicator framework.

[CR24] Falkenmark M (2004). Towards integrated catchment management: Opening the paradigm locks between hydrology, ecology and policy-making. International Journal of Water Resources Development.

[CR25] Goepel, K. D. (2013). Implementing the Analytic Hierarchy Process as a standard method for multi-criteria decision making in corporate enterprises: A New AHP Excel template with multiple inputs. In *Proceedings of the international symposium on the analytic hierarchy process, Kuala Lumpur*, June 23-26, 2013, 1–10.

[CR26] González-Bravo R, Marques MC, Bezerra MO, Coutinho B, Castillo JLD, Vollmer D, Ramirez O, Mahlknecht J (2019). Urban sustainability: Analyzing the water-energy nexus in the Guandu river basin, Rio de Janeiro, Brazil. Energy Reports.

[CR27] Hooper B (2010). River basin organization performance indicators: Application to the Delaware River basin commission. Water Policy.

[CR28] Huitema D, Meijerink S (2017). The politics of river basin organizations: Institutional design choices, coalitions, and consequences. Ecology and Society.

[CR29] Jacobson M, Meyer F, Oia I, Reddy P, Tropp H (2013). User’s guide on assessing water governance.

[CR30] Kaufmann D, Kraay A, Mastruzzi M (2010). The worldwide governance indicators: A summary of methodology, data and analytical issues. World Bank Policy Research.

[CR31] Knieper C, Holtz G, Kastens B, Pahl-Wostl C (2010). Analysing water governance in heterogeneous case studies: Experiences with a database approach. Environmental Science & Policy.

[CR58] Liu X, Souter NJ, Wang RY, Vollmer D (2019). Aligning the freshwater health index indicator system against the transboundary water governance framework of Southeast Asia’s Sesan, Srepok, and Sekong River Basin. Water.

[CR32] McDonnell RA (2008). Challenges for integrated water resources management: How do we provide the knowledge to support truly integrated thinking?. International Journal of Water Resources Development.

[CR33] Musacchio A, Re V, Mas-Pla J, Sacchi E (2020). EU Nitrates Directive, from theory to practice: Environmental effectiveness and influence of regional governance on its performance. Ambio.

[CR34] Norman ES, Dunn G, Bakker K, Allen DM (2013). Water security assessment: Integrating governance and freshwater indicators. Water Resources Management.

[CR59] Novakowski N, Wellar B (2008). Using the Delphi technique in normative planning research: Methodological design considerations. Environment and Planning A.

[CR35] Olsson L, Jerneck A (2018). Social fields and natural systems: Integrating knowledge about society and nature. Ecology and Society.

[CR36] Ostovar AL (2019). Investing upstream: Watershed protection in Piura, Peru. Environmental Science & Policy.

[CR37] Özerol G, Vinke-De Kruijf J, Brisbois MC, Flores CC, Deekshit P, Girard C, Knieper C, Mirnezami SJ (2018). Comparative studies of water governance: A systematic review. Ecological Society.

[CR38] Pahl-Wostl C (2019). The role of governance modes and meta-governance in the transformation towards sustainable water governance. Environmental Science & Policy.

[CR39] Pahl-Wostl C, Jeffrey P, Isendahl N, Brugnach M (2010). Maturing the new water management paradigm: Progressing from aspiration to practice. Water Resources Management.

[CR40] Pires A, Morato J, Peixoto H, Botero V, Zuluaga L, Figueroa A (2017). Sustainability assessment of indicators for integrated water resources management. Science of the Total Environment.

[CR41] Rogers, B. P. and A. W. Hall. 2003. *Effective water governance*. global water partnership Technical Committee (TEC) Background Paper Number 7. ISBN: 91-974012-9-3. Global Water Partnership, Sweden.

[CR42] Rouillard JJ, Spray CJ (2017). Working across scales in integrated catchment management: Lessons learned for adaptive water governance from regional experiences. Regional Environmental Change.

[CR43] Saaty T, Figueira J, Greco S, Ehrgott M (2005). The analytic hierarchy and analytic network processes for the measurement of intangible criteria and for decision-making. Multiple criteria decision analysis: State of the art surveys.

[CR44] Schneider F, Bonriposi M, Graefe O, Herweg K, Homewood C, Huss M, Kauzlaric M, Liniger H (2014). Assessing the sustainability of water governance systems: The sustainability wheel. Journal of Environmental Planning and Management.

[CR45] Sharpe A (2004). Literature review of frameworks for macro-indicators.

[CR60] Souter NJ, Shaad K, Vollmer D, Regan HM, Farrell TA, Arnaiz M, Meynell PJ, Cochrane TA (2020). Using the freshwater health index to assess hydropower development scenarios in the Sesan, Srepok and Sekong River Basin. Water.

[CR46] Stefano LD (2010). International initiatives for water policy assessment: A review. Water Resources Management.

[CR47] Stern, M., and M. Echavarria. 2013. Investments in watershed services for Moyobamba on Subwatersheds of the Alto Mayo, Department of San Martín, Peru. Peru Investments in Watershed Services Series. Washington, DC: Forest Trends.

[CR48] Sullivan GM, Artino AR (2013). Analyzing and interpreting data from Likert-type scales. Journal of Graduate Medical Education.

[CR49] Tortajada C (2010). Water governance: Some critical issues. International Journal of Water Resources Development.

[CR50] UNEP-DHI (UN-Environment DHI Centre on Water and Environment). 2018. IWRM Data Portal, http://iwrmdataportal.unepdhi.org/index.html.

[CR51] Vaske JJ, Beaman J, Sponarski CC (2017). Rethinking internal consistency in Cronbach’s alpha. Leisure Sciences.

[CR52] Vollmer D, Regan HM, Andelman SJ (2016). Assessing the sustainability of freshwater systems: A critical review of composite indicators. Ambio.

[CR53] Vollmer D, Shaad K, Souter NJ, Farrell T, Dudgeon D, Sullivan CA, Fauconnier I, MacDonald GM (2018). Integrating the social, hydrological and ecological dimensions of freshwater health: The Freshwater Health Index. Science of the Total Environment.

[CR54] Wesselink A, Kooy M, Warner J (2017). Socio-hydrology and hydrosocial analysis: Toward dialogues across disciplines. Wiley Interdisciplinary Reviews: Water.

[CR55] Wiek A, Larson KL (2012). Water, people, and sustainability: A systems framework for analyzing and assessing water governance regimes. Water Resources Management.

[CR56] Wilde, A., S. Narang,, M. Laberge,, and L. Moretto. 2009. A Users’ Guide to Measuring Local Governance. *UNDP Oslo Governance Centre*.

[CR57] Woodhouse P, Muller M (2017). Water governance: An historical perspective on current debates. World Development.

