# Supplementary material for: Can we take the pulse of environmental governance the way we take the pulse of nature? Applying the Freshwater Health Index in Latin America
Source: Ambio. 2020 Nov 15;50(4):870–83. doi: 10.1007/s13280-020-01407-8 (PMC7982359; doi:10.1007/s13280-020-01407-8)
Supplement: Supplementary file 1 — Supplementary material 1 (PDF 325 kb) [file 13280_2020_1407_MOESM1_ESM.pdf]

Ambio

Electronic Supplementary Material

This supplementary material has not been peer reviewed

Title: **Can we take the pulse of environmental governance the way we take the pulse of nature? Applying the Freshwater Health Index in Latin America**

Derek Vollmer, Maíra Ometto Bezerra, Natalia Acero Martínez, Octavio Rodríguez Ortiz, Ivo Encomenderos, Maria Clara Marques, Lina Serrano-Durán, Isabelle Fauconnier, Raymond Yu Wang

Appendix A. Questionnaire used for the survey of water governance (English version)

The Freshwater Health Index is an analytical tool developed by Conservation International and partners to promote freshwater security and the sustainable management of freshwater ecosystems. It provides a comprehensive assessment of freshwater ecosystems along three dimensions--ecosystem vitality, ecosystem services, and governance—with a goal of linking science, policy and practice.

This survey is designed to gather information for the governance assessment, and aims to understand the views of different stakeholders from your basin on the coordinating mechanisms, participatory processes, governance effectiveness, and long-term planning within the region. **Your response to the survey and all questions is voluntary.** Your valuable advice will provide a basis for a comprehensive assessment of the state of the current governance system within the basin. Your answers will remain anonymous, but we will ask for your opinions (not the views of your institution) as well as basic identifiers of your country and affiliation. The information collected will only be used for research purposes, personal data will be kept confidential. Thank you for your cooperation and help!

Your current affiliation

☐ Local government ☐ Provincial government ☐ NGO

☐ Research/academia ☐ National government ☐ Industry

☐ Other (please specify): ______________________________

Framework for Basin Management (1 of 12)

Integrated water resources management is a guiding framework for coordinating both development and management of all resources within a basin, to maximize welfare without compromising ecological sustainability. In some cases a single agency, such as a river basin authority, is responsible for coordinating and overseeing these functions; the questions below focus on the specific functions as managed within your country, regardless of whether they are all carried out by the same agency.

Based on your own knowledge of the current situation, please evaluate the degree to which the following functions are being fulfilled throughout the basin. Provide a rating between 1 and 5 following the criteria below as a guide. Please skip any items which you do not feel qualified to answer.

Rating	Criteria
1	Function is almost never satisfactory (without conflicts among stakeholder groups)
2	Function is rarely satisfactory
3	Function is sometimes (~50%) satisfactory
4	Function is often satisfactory
5	Function is almost always satisfactory

A) **Policies and actions to advance water resource development and management are coordinated.**
For example, but not limited to, if there is river basin organization or commission, how effective is it in coordinating the different sectoral agencies, levels of government (e.g., national, provincial, local), and private interests when developing integrated development plans for the basin?

☐ 1 ☐ 2 ☐ 3 ☐ 4 ☐ 5

B) **Infrastructure such as dams, reservoirs, and treatment plants are centrally managed or coordinated.**
Examples include, but are not limited to: dam operators communicating the timing and volume of reservoir releases, or assessing cumulative impacts of dams.

☐ 1 ☐ 2 ☐ 3 ☐ 4 ☐ 5

C) **Financial resources are mobilized to support water resource development and management needs.**
Examples include, but are not limited to: cost-sharing for common projects, or collecting user fees/taxes.

☐ 1 ☐ 2 ☐ 3 ☐ 4 ☐ 5

D) **Ecosystems conservation priorities are developed and actions implemented.**
Examples include, but are not limited to: protecting forested watersheds, maintaining wetland connectivity, or developing a biodiversity action plan for aquatic species.

☐ 1 ☐ 2 ☐ 3 ☐ 4 ☐ 5

Rules for resource use (2 of 12)

Clear and enforceable rules are generally recognized as a precondition to efficient use of scarce resources, and as a means of resolving conflicts. These rules encompass various uses and users of water, and can be both formal (i.e., legislated by a government body) or informal rules administered by communities.

Based on your own knowledge of the current situation, please evaluate the quality and stakeholders' understanding of rules pertaining to the various types of resource use. Provide a rating between 1 and 5 following the criteria below as a guide. Please skip any items which you do not feel qualified to answer.

Rating	Criteria
1	Rules are very poorly articulated and/or understood or do not exist
2	Rules are poorly articulated and/or understood
3	Rules are acceptably articulated and/or understood
4	Rules are well articulated and/or understood
5	Rules are very well articulated and/or understood

A) **Quality and clarity of rules for allocating water among different sectors (e.g., municipal, industrial, agricultural)**
Examples include, but are not limited to: prioritizing water according to use, or limits on the timing and amount that can be withdrawn.

☐ 1 ☐ 2 ☐ 3 ☐ 4 ☐ 5

B) **Quality and clarity of rules for allocating water among administrative jurisdictions (e.g., cities, provinces, countries)**
Examples include, but are not limited to: determining withdrawals between provinces, or setting minimum flow requirements for rivers that cross administrative boundaries.

☐ 1 ☐ 2 ☐ 3 ☐ 4 ☐ 5

C) **Quality and clarity of rules for groundwater abstraction**
Examples include, but are not limited to: guidelines regarding the depth of wells, or amount of water that can be withdrawn within a certain time period.

☐ 1 ☐ 2 ☐ 3 ☐ 4 ☐ 5

D) **Quality and clarity of rules for wastewater handling and water pollution emissions**
Examples include, but are not limited to: guidelines regarding the discharge of wastewater (e.g. pollutant concentrations, volume, temperature, time of release) back into water bodies.

☐ 1 ☐ 2 ☐ 3 ☐ 4 ☐ 5

E) **Quality and clarity of rules for managing land use (including aquaculture) to safeguard water resources**
Examples include, but are not limited to: guidelines regarding soil management practices, the amount of forested land cover in watersheds, or the volume of runoff allowed for a given plot of land.

☐ 1 ☐ 2 ☐ 3 ☐ 4 ☐ 5

F) **Quality and clarity of rules for freshwater fisheries**
Examples include, but are not limited to: guidelines on catch limits, protected species, or fishing methods.

☐ 1 ☐ 2 ☐ 3 ☐ 4 ☐ 5

Incentives and regulations (3 of 12)

Various management tools, from conventional regulations to market-based instruments can be applied within a governance system. Having a variety of tools offers opportunities to increase the efficiency of interventions (e.g., cost per unit outcome) or lead to a more equitable distribution of benefits.

Based on your own knowledge of the current situation, please evaluate the development of the following management tools. Provide a rating between 1and 5 following the criteria below as a guide. Please skip any items which you do not feel qualified to answer.

Rating	Criteria
1	Instrument does not exist or is in earliest stage of discussion
2	Instrument is under development, e.g. guidelines have been promulgated
3	Instrument has been developed and is being piloted, but guidelines are subject to refinement
4	Instrument is fully developed but use is not yet standardized
5	Instrument is fully developed and a standard practice

A) **Environmental and social impact assessments for all major water projects, regardless of funding source, are carried out prior to decisions being taken**
Examples include, but are not limited to: environmental impact assessment (EIA) that is submitted to a government body for evaluation.

☐ 1 ☐ 2 ☐ 3 ☐ 4 ☐ 5

B) **Existence of financial incentives for environmental stewardship**
Examples include, but are not limited to: mechanisms for providing payments for watershed services provided by upstream stakeholders (e.g., farmers, forest managers, local governments).

☐ 1 ☐ 2 ☐ 3 ☐ 4 ☐ 5

C) **Existence of market-based exchange schemes**
Examples include, but are not limited to: tradeable water rights, wetland mitigation banking, or pollutant trading.

☐ 1 ☐ 2 ☐ 3 ☐ 4 ☐ 5

D) **Existence of honorary recognition programs**
Examples include, but are not limited to: publishing lists of industries with good environmental performance, or awards for local governments exhibiting good water stewardship.

☐ 1 ☐ 2 ☐ 3 ☐ 4 ☐ 5

E) **Existence of land use zoning policy**
Examples include, but are not limited to: requirements for riparian buffers, floodplain development, or forested catchment zones.

☐ 1 ☐ 2 ☐ 3 ☐ 4 ☐ 5

Technical capacity (4 of 12)

Lack of local capacity is often cited as an impediment to a variety of issues in resource management. Here we are referring specifically to people employed in areas of water resource management, service delivery, monitoring and enforcement, and related research, but excluding international consultants.

Based on your own knowledge of the current situation, please evaluate the quality of human resources in water resource development and management in the basin. Provide a rating between 1 and 5 following the criteria below as a guide. Please skip any items which you do not feel qualified to answer.

Rating	Criteria
1	Level is very unsatisfactory
2	Level is unsatisfactory
3	Level is satisfactory
4	Level is very satisfactory
5	Level is extremely satisfactory

A) Number of staff (including local consultants) to fulfill necessary functions
Examples include, but are not limited to: backlogs (work waiting to be done) in a particular agency, or open positions remaining vacant due to lack of candidates.

☐ 1 ☐ 2 ☐ 3 ☐ 4 ☐ 5

B) Staff have sufficient expertise to fulfill necessary functions
Examples include, but are not limited to: hydrologists to evaluate a proposed dam, or fish ecologists to assess fisheries stocks.

☐ 1 ☐ 2 ☐ 3 ☐ 4 ☐ 5

C) Opportunities for professional training and certification
Examples include, but are not limited to: financial support or time allocated for continuing education courses related to improving technical skills.

☐ 1 ☐ 2 ☐ 3 ☐ 4 ☐ 5

Financial capacity (5 of 12)

Water resource development and management is often chronically under-financed, particularly for services that do not generate revenue, such as ecosystem protection. Although financial capacity can be measured directly as a function of existing allocations relative to estimated budget needs, qualitative information is also useful in providing insights and identifying priorities.

Based on your own knowledge of the current situation, please evaluate the quality of human resources in water resource development and management in the basin. Provide a rating between 1 and 5 following the criteria below as a guide. Please skip any items which you do not feel qualified to answer.

Rating	Criteria
1	Level is very unsatisfactory
2	Level is unsatisfactory
3	Level is satisfactory
4	Level is very satisfactory
5	Level is extremely satisfactory

A) Level of investment in water supply development
Examples include, but are not limited to: financial resources for building and maintaining reservoirs or irrigation systems.

☐ 1 ☐ 2 ☐ 3 ☐ 4 ☐ 5

B) Level of investment in service delivery systems
Examples include, but are not limited to: financial resources for building and maintaining water distribution networks (i.e. piped supply) or household wells.

☐ 1 ☐ 2 ☐ 3 ☐ 4 ☐ 5

C) Level of investment in wastewater handling and treatment
Examples include, but are not limited to: financial resources for building and maintaining community sanitation facilities, or treatment systems to process waste water.

☐ 1 ☐ 2 ☐ 3 ☐ 4 ☐ 5

D) Level of investment in ecosystem conservation and rehabilitation
Examples include, but are not limited to: financial resources for protecting wetlands to mitigate flood risk, remediating impaired streams, or rehabilitating fisheries stocks.

☐ 1 ☐ 2 ☐ 3 ☐ 4 ☐ 5

E) Level of investment in monitoring and enforcement
Examples include, but are not limited to: financial resources for evaluating EIAs, collecting environmental data, inspecting facilities, and enforcing regulations.

☐ 1 ☐ 2 ☐ 3 ☐ 4 ☐ 5

Information and knowledge (6 of 12)

Sound water governance requires information on a wide range of topics and from many sources. Even in cases where data and information are abundant, if they are not made accessible (across agencies, with citizens, etc.) then they are less likely to aid in wise decision making.

Based on your own knowledge of the current situation, please evaluate the accessibility of information (including data on water quantity and quality, planning documents, and financial information), along with its quality of coverage and transparency (ability to be traced to the source). Provide a rating between 1 and 5 following the criteria below as a guide. Please skip any items which you do not feel qualified to answer.

Rating	Criteria
1	Almost never satisfactory
2	Rarely satisfactory
3	Sometimes (~50%) satisfactory
4	Often satisfactory
5	Almost always satisfactory

A) **Information is accessible to interested stakeholders**
Examples include, but are not limited to: reports made freely available through a website, or data available upon request to the agency with the information.

☐ 1 ☐ 2 ☐ 3 ☐ 4 ☐ 5

B) **Information meets expected quality standards, in terms of frequency, level of detail, and subjects of interest to stakeholders**
Examples include, but are not limited to: time series data on streamflow, water levels, or water quality for specific locations within the basin.

☐ 1 ☐ 2 ☐ 3 ☐ 4 ☐ 5

C) **Information is transparently sourced**
Examples include, but are not limited to: methods used to collect data are documented, or authors (source) of these data are clearly identified.

☐ 1 ☐ 2 ☐ 3 ☐ 4 ☐ 5

D) All available information is routinely applied in decision-making
Examples include, but are not limited to: modifying an infrastructure project based on EIA results, or adjusting fisheries management guidelines based on fish catch data.

☐ 1 ☐ 2 ☐ 3 ☐ 4 ☐ 5

Engagement in decision-making processes (7 of 12)

Stakeholder engagement encompasses the process by which any person or group with an interest in a water-related topic can be involved in decision-making and implementation. It is associated with improved information transfer, better targeted and more equitable plans and policies, improved transparency and accountability, and reduced conflict.

Based on your own knowledge of the current situation, please evaluate the degree to which all stakeholders have a voice within the cycle of policy and planning for water resources development and management. Provide a rating between 1 and 5 following the criteria below as a guide. Please skip any items which you do not feel qualified to answer.

Rating	Criteria
1	Process as described almost never occurs
2	Process as described rarely occurs
3	Process as described sometimes (~50%) occurs
4	Process as described often occurs
5	Process as described almost always occurs

A) **All relevant stakeholders have been identified and notified when considering major decisions**
Examples include, but are not limited to: mapping and notifying stakeholders affected by a proposed water supply infrastructure project.

☐ 1 ☐ 2 ☐ 3 ☐ 4 ☐ 5

B) **Stakeholders are able to provide comments prior to major decisions being taken**
Examples include, but are not limited to: consultation meetings or an information solicitation period where stakeholders have an opportunity to provide input regarding a policy or project.

☐ 1 ☐ 2 ☐ 3 ☐ 4 ☐ 5

C) Decisions are responsive to stakeholders' participation
Examples include, but are not limited to: processes for reaching joint agreements among a group of stakeholders prior to approval of a major policy or project, or projects being revised subsequent to stakeholder feedback.

☐ 1 ☐ 2 ☐ 3 ☐ 4 ☐ 5

Enforcement and compliance (8 of 12)

In many societies, there is a gap between laws and their actual enforcement, reflecting either insufficient capacity or a lack of accountability. Enforcement and compliance can be ensured through fines, incentives, or social pressure, but weak enforcement leads to missing goals and undermines confidence in the system.

Based on your own knowledge of the current situation, please evaluate the adequacy of enforcement of existing regulations and agreements for the following issue areas throughout the basin. Provide a rating between 1 and 5 following the criteria below as a guide. Please skip any items which you do not feel qualified to answer.

Rating	Criteria
1	Enforcement is very poor or no guidelines (formal or informal) exist
2	Enforcement is poor
3	Enforcement is acceptable
4	Enforcement is good
5	Enforcement is very good

A) **Surface water abstraction guidelines are enforced**
Examples include, but are not limited to: industries effectively restricted from withdrawing more than a specified amount of surface water, or farmers sanctioned for withdrawals during the dry season.

☐ 1 ☐ 2 ☐ 3 ☐ 4 ☐ 5

B) **Groundwater abstraction guidelines are enforced**
Examples include, but are not limited to: farmers or industries restricted from pumping more than a specified amount of groundwater.

☐ 1 ☐ 2 ☐ 3 ☐ 4 ☐ 5

C) Flow requirement guidelines are enforced
Examples include, but are not limited to: dam operators meeting the expectations of downstream water users, to meet environmental flows, human water needs, and/or flood protection.

☐ 1 ☐ 2 ☐ 3 ☐ 4 ☐ 5

D) **Water quality guidelines are enforced**
Examples include, but are not limited to: industries and communities complying with requirements related to pollutant discharges, or non-negotiable fines are levied on violators.

☐ 1 ☐ 2 ☐ 3 ☐ 4 ☐ 5

E) Land use guidelines are enforced
Examples include, but are not limited to: environmentally sensitive zones (e.g., catchment forests and wetlands) being protected from development or degradation.

☐ 1 ☐ 2 ☐ 3 ☐ 4 ☐ 5

Distribution of benefits from ecosystem services (9 of 12)

*Equity is an important issue in water resource management, most closely associated with access to safe water and sanitation. Here we extend the concept to include <u>all benefits</u> from ecosystem services in the basin (**water and sanitation, fisheries, flood mitigation, water quality maintenance, disease regulation, and cultural services**).*

Based on your own knowledge of the current situation, please evaluate quality of outcomes, in terms of their share of benefits from water resources, for the following groups of stakeholders (groupings may overlap). Provide a rating between 1 and 5 following the criteria below as a guide. <u>Please skip any items which you do not feel qualified to answer</u>.

Rating	Criteria
1	Their share of benefits is almost never adequate
2	Their share of benefits is rarely adequate
3	Their share of benefits is sometimes (~50%) adequate
4	Their share of benefits is often adequate
5	Their share of benefits is almost always adequate

A) **Economically vulnerable populations benefit from ecosystem services**
Examples include, but are not limited to: poor households' access to improved water supply sources at a reasonable cost, protection from inland flood risks, or rural compared to urban populations' benefits.

☐ 1 ☐ 2 ☐ 3 ☐ 4 ☐ 5

B) Indigenous people benefit from ecosystem services
Examples include, but are not limited to: exercising customary rights related to water, including for consumptive as well as cultural uses, or maintaining traditional fisheries.

☐ 1 ☐ 2 ☐ 3 ☐ 4 ☐ 5

C) Women and girls benefit from ecosystem services
Examples include, but are not limited to: amount of time collecting water for households, or provision of latrines for females.

☐ 1 ☐ 2 ☐ 3 ☐ 4 ☐ 5

D) **Resource-dependent communities benefit from ecosystem services**
Examples include, but are not limited to: fishermen and smallholder farmers' incomes compared to other economic sectors.

☐ 1 ☐ 2 ☐ 3 ☐ 4 ☐ 5

Water-related conflict (10 of 12)

Tensions among stakeholders are expected when there is competition for scarce resources such as water services. An effective governance system should prevent tensions from escalating into conflicts, here defined as a difference that prevents agreement, and therefore delays or undermines a decision taken with the basin.

Based on your own knowledge of the current situation, please evaluate the frequency of conflicts occurring over the past three years regarding water-related issues. Provide a rating between 1 and 5 following the criteria below as a guide. Please skip any items which you do not feel qualified to answer.

Rating	Criteria
1	Conflicts almost always occur
2	Conflicts often occur
3	Conflicts sometimes occur
4	Conflicts rarely occur
5	Conflicts almost never occur

A) **Frequency of conflict due to overlapping jurisdictions (e.g., between national governments in transboundary systems, provincial and national government, or between sectoral agencies)**
Examples include, but are not limited to: disputes between the local environmental bureau and a national ministry about authority within a floodplain, or between agencies in managing agricultural pollution.

☐ 1 ☐ 2 ☐ 3 ☐ 4 ☐ 5

B) **Frequency of conflict about water rights allocation**
Examples include, but are not limited to: disputes about how water is allocated between two municipalities, or between agricultural and industrial users.

☐ 1 ☐ 2 ☐ 3 ☐ 4 ☐ 5

C) **Frequency of conflict about access**
Examples include, but are not limited to: disputes about having access to safe water and sanitation, or the costs of such access.

☐ 1 ☐ 2 ☐ 3 ☐ 4 ☐ 5

D) Frequency of conflict regarding the siting of infrastructure
Examples include, but are not limited to: disputes about reservoir development and resettlement plans for residents and land owners, or downstream impacts to fisheries or water users.

☐ 1 ☐ 2 ☐ 3 ☐ 4 ☐ 5

E) Frequency of conflict over water quality and other downstream negative impacts
Examples include, but are not limited to: disputes between upstream and downstream stakeholders about dry season flows or pollution concentrations.

☐ 1 ☐ 2 ☐ 3 ☐ 4 ☐ 5

Monitoring mechanisms (11 of 12)

Policy and planning decisions about water resources management are ideally based on sound data and information, which must be collected on a regular basis. Monitoring entails costs and so data collection should be based on needs and assessed relative to resource constraints, where a comparatively wealthy basin might invest in higher spatial and temporal coverage of information.

Based on your own knowledge of the current situation, please evaluate the degree to which different types of data are being collected, analyzed, and used to inform decisions in the basin. Provide a rating between 1 and 5 following the criteria below as a guide. Please skip any items which you do not feel qualified to answer.

Rating	Criteria
1	Data are very poorly monitored or not monitored at all
2	Data are poorly monitored
3	Data are acceptably monitored
4	Data are well monitored
5	Data are very well monitored

A) Overall standard of water quantity monitoring
Examples include, but are not limited to: streamflow being regularly measured, estimated, or modeled in the basin

☐ 1 ☐ 2 ☐ 3 ☐ 4 ☐ 5

B) Overall standard of water quality monitoring
Examples include, but are not limited to: water quality samples taken from water bodies and measured, or water quality being modeled based on data related to discharge of pollutants.

☐ 1 ☐ 2 ☐ 3 ☐ 4 ☐ 5

C) **Overall standard of biological and ecological monitoring**
Examples include, but are not limited to: surveillance undertaken to assess aquatic species (e.g., harvested, threatened, invasive) populations or communities (e.g. macroinvertebrates).

☐ 1 ☐ 2 ☐ 3 ☐ 4 ☐ 5

D) **Overall standard of monitoring access to, and use of, water**
Examples include, but are not limited to: household surveys administered to estimate the coverage of access to improved water and sanitation sources, or estimates of farmers' groundwater extraction.

☐ 1 ☐ 2 ☐ 3 ☐ 4 ☐ 5

Comprehensive planning and adaptive management (12 of 12)

Comprehensive planning here is defined as addressing water quantity and quality, surface and groundwater, land use and ecology, and multiple stakeholders' objectives. Adaptive management refers to the ability to handle changes, unintended consequences, or surprises to the water resource system.

*Based on your own knowledge of the current situation, please evaluate the degree to which **comprehensive** planning at the basin (or sub-basin) scale is taking place. Provide a rating between 1 and 5 following the criteria below as a guide. <u>Please skip any items which you do not feel qualified to answer</u>.*

Rating	Criteria
1	Process is almost never comprehensive (or does not occur at all)
2	Process is rarely comprehensive
3	Process is sometimes (~50%) comprehensive
4	Process is often comprehensive
5	Process is almost always comprehensive

A) **A shared vision is established and used to set objectives and guide future development**
Examples include, but are not limited to: goals for improvement are jointly established by multiple stakeholders, or a process is in place for developing local water plans that inform higher-level (provincial or national) plans.

☐ 1 ☐ 2 ☐ 3 ☐ 4 ☐ 5

B) **The existence and use of strategic planning mechanisms**
Examples include, but are not limited to: basin-specific spatial plans or management plans that guide investments and policy, or climate change adaptation plans.

☐ 1 ☐ 2 ☐ 3 ☐ 4 ☐ 5

C) **The existence and use of an adaptive management framework**
Examples include, but are not limited to: updating plans to reflect new knowledge or changing economic development priorities, or to address issues such as climate change.

☐ 1 ☐ 2 ☐ 3 ☐ 4 ☐ 5
